# Pastoral ministry and persons with disabilities: The case of the Apostolic Faith Mission in Zimbabwe

**DOI:** 10.4102/ajod.v8i0.431

**Published:** 2019-02-20

**Authors:** Nomatter Sande

**Affiliations:** 1 Department of Religion and Social Transformation, University of KwaZulu-Natal, South Africa; 2 Apostolic Faith Mission International Ministries, Leicester, United Kingdom

## Abstract

**Background:**

The Persons with Disability (PWD) are the minority group dehumanized in the church. The subject of disability is complicated because of the impact of the Judeo-Christian teachings. The Apostolic Faith Mission (AFM) in Zimbabwe is a leading Pentecostal church with a pastoral ministry theology which emphasises divine healing, miracles, signs and wonders. Thus, the space of PWD and how the PWD either connects or benefits from this Pentecostal heritage is a critical gap in this study.

**Objectives:**

The objective of this study was to explore the construction of disability through the practices and processes of the pastoral ministry in the AFM.

**Method:**

This study followed qualitative research and used the social model of disability as theoretical framework. The data were collected from 26 participants who are PWD and pastors using in-depth interviews, focus groups and participant observations.

**Results:**

The results showed the AFM pastoral practices created invisible barriers that militate against PWD. Thus, the pastoral ‘divine solutions’ and ‘triumphalist messages and teachings’ are ‘prescriptive’ and ineffective in reducing ‘the plight of PWD in Zimbabwe’.

**Conclusion:**

The study concludes that the pastoral ministry should be ‘one efficient vehicle’ with which the church can care for and ‘transform persons with disabilities’. Pastors should break the glass ceiling by expecting pastors to minister better and more effectively creating a safe space for persons with disabilities. A caring community should be the nature of both the AFM and the pastoral ministry responsible for meeting the needs of the persons with disabilities.

## Introduction

In the Apostolic Faith Mission (AFM) in Zimbabwe, the Pentecostal theology is perceived as having become a source of hope and refuge for the suffering populace in Zimbabwe. Although there are harmful elements such as fraud, fake miracles and gullibility attached to the Pentecostal religious landscape in Zimbabwe (Chitando, Gunda & Kügler 2013; Gunda 2012; Maposa & Marongwe 2015), Pentecostalism has attempted to address the wants and needs of the suffering people of Zimbabwe. As such, employing divine solutions to the challenges has been prescribed to those seeking help. The package and tools for breakthroughs include, but are not limited to, prayer, believing in the Word of God, prophecy and financial giving or seeding to both the church and the pastors. The role and significance of the church and pastoral ministry has increased in the lives of the people. The AFM in Zimbabwe has been regarded by its adherence as a spiritual home for both non-disabled persons and persons with disabilities. Regardless of this, persons with disabilities tend to experience exclusion and stigmatisation when it comes to integration into the mainstream of faith-based organisations. No significant, deliberate theologies are targeting the plight of persons with disabilities, and as such, churches have created invisible barriers and practices that disable persons with disabilities. Accordingly, the purported ‘divine solutions’ and ‘triumphalist messages and teaching’ used are ‘prescriptive’ and ineffective in reducing ‘the disability prevalence’ in Zimbabwe. Statistics show that the disability prevalence in Zimbabwe is at 7%, amounting to 900 000 individuals (UNICEF 2013).

## Definition of pastoral care

Pastoral ministry is one of the most potent tools in the church, responsible for communicating ideas, perspectives and attitudes. Pastors are respected and given a sacred position within the religious community. Pembroke (2009:1) argued that personal ministry to individuals and family is a critical component of pastoral ministry and the focus of worship is theocentric, and God is both the object and subject of worship. As such, this article argues that pastoral ministry ‘is one efficient vehicle’ with which the church can care for and ‘help persons with disabilities’. A ‘caring community’ defines the nature of the AFM in Zimbabwe and pastoral ministry and is the condition for the proposed transformation. Magezi (2016:1) defines pastoral care as a caring ministry of the religious community. Pastoral ministry is broader than pastoral care and means ‘church or parish’ ministry. The term ‘pastoral’ signifies shepherding the vulnerable (McClure 2012:269) and pastoral care helps in building relationships in the church. Accordingly, this suggests that the pastors within the AFM in Zimbabwe are essential to meeting the needs of both the non-disabled and persons with disabilities. Further, pastoral counselling is a helping relationship with an expressly concurred, firm encouragement of relationships. Thus, pastoral ministry and care bring to the fore theological perspectives of the image of God, love, compassion and social justice concerning persons with disabilities. As such, this article connects vital themes responsible for the construction of disability through the practices and processes of the pastoral ministry in the AFM in Zimbabwe.

## Theoretical framework and methodology

This article uses the social model of disability as the theoretical framework. Disability is trans-disciplinary, permeating through medicine and theology. The social model of disability defines disability as a social construct (Devlieger & De Coster 2009; Haihambo & Lightfoot 2010; Wendell 1996). In this case, the social model of disability argues that disability is something that is created by barriers that exist within the society. Accepting that disability is socially constructed automatically raises questions that will help this article to analyse how the AFM in Zimbabwe both constructs disability and responds to the needs of persons with disabilities. This article proposes that pastoral ministry in the AFM disables the persons with disabilities. Thus, this article uses the social model of disability to expose these social barriers. This article uses the social model of disability not only to aid in analysing the construction of disability within the AFM in Zimbabwe but also to point towards strategies for addressing the challenges.

Methodologically, this article falls under qualitative research. The content of this article is an excerpt from the author’s PhD thesis, ‘Pastoral ministry to persons with disabilities: A critical investigation of how AFM Church can be a safe space for meeting the needs of people with disabilities (PWD) in Harare Zimbabwe.’^1^ Data collection was done from in-depth interviews, focus groups and participant observations. The total number of participants was 26, and the sample consisted of persons with disabilities and pastors. The variables for the recruitment strategy involved the nature of the disability, age, sex and place of residence. The author used three tools to gather information for this article: in-depth interviews, focus groups and participant observations. The in-depth interviews were with pastors (non-disabled). The interviews gathered information about the pastoral ministry’s response to the persons with disabilities in the AFM. The sample for the focus groups consisted of persons with a physical disability. Because of the complexity of the disability category, the author relied heavily on how the participants would describe the nature of their disability. The author used three focus groups: The first focus group consisted of the youths from 16 to 21 years; the second group consisted of both men and women from 21 to 40 years; and the third group consisted of elder men and women from 41 and above. The focus groups explored the lived experiences of the persons with disabilities within the AFM in Zimbabwe. The author used participant observation and maintained a professional distance to observe and record data. Being an ordained pastor in the AFM in Zimbabwe for more than 10 years, the author used his experiential knowledge and critically observed three AFM in Zimbabwe national conferences. The author focused on how persons with disabilities were treated and made meaning of the pastors’ response to persons with disabilities during the AFM corporate liturgy.

### Ethical considerations

Ethical clearance was obtained from the University of KwaZulu-Natal and the AFM gave permission to conduct the research. The author ensured that the participants participated willingly in the study, and from an informed position. Permission was sought to publish the research results from the participants and the author ensured the participants aware of the purpose and objectives of the study. The author emphasised that participants could withdraw from the study at any time, should they feel to do so, without stating any reason for that choice. It was made clear to participants that there was no financial benefit from this study but the participants would benefit from the contribution this research will make towards transforming the lives of PWD. The author ensured confidentiality and protection from harm for each participant by using a coding system.

## An overview of the Apostolic Faith Mission in Zimbabwe

The AFM in Zimbabwe falls within the African Pentecostal landscape. The Azusa Street Revival in Los Angeles, California, happened in 1906 and is believed to be the outburst for the Pentecostal movement. The emergence of AFM in Zimbabwe is associated with the migrant worker who brought the movement from South Africa in 1915 (Hwata 2005). The AFM in Zimbabwe has about 2.3 million members in Zimbabwe (Machingura & Chivasa 2016:13; Togarasei, 2016; Sande 2017a). The AFM in Zimbabwe is regarded as a home and spiritual hub for believers. The history, prominent beliefs and liturgy of the AFM in Zimbabwe help to put this article in context. Musoni (2013:76) argued that Pentecostals seek to re-establish the miracles and Holy Spirit baptisms of the New Testament. Pentecostalism relies on the Holy Spirit and gifts of the Holy Spirit, and a longing for remarkable encounters, healings and deliverances (Anderson 2004). Pentecostals yearn for the infilling of the Holy Spirit which is believed to bring the power of the Holy Ghost in the daily lives of the believers as attested by Acts 2:8. Pentecostals create room for divine manifestation and the ‘unexpected to happen’ (Smith 2010:39). Also, Dayton (1994:26) argued that the Pentecostal experience goes with the supernatural elements of the Holy Spirit, showing that divine healing is both a gift of God and sign of the presence of God in believer’s life.

Divine healing and restorative miracles are believed to be prominent in the AFM in Zimbabwe. The AFM in Zimbabwe believes that the blind receive sight, the lame will walk and the dumb will speak. Burger et al. (1997:167) argued that one of the reasons people joined the AFM in Zimbabwe was because of healing they received or that was testified to them. As such, people give testimonies about how God will have performed miracles in their lives. Thus, testimonies are Pentecostalism’s most profound characteristics. Testimonies of what God is doing in the life of the believers are key in AFM in Zimbabwe. Hollenweger (1999:36–39) contends that ‘oral liturgy’ is a narrative theology that incorporates, dreams, visions, healings and the interests of the entire group in worship. Thus, Pentecostals have a culture that communicates orally to develop a theology. The roots of oral culture make testimonies prominent in Pentecostalism, and these are formulated through stories and not abstract propositions (Cartledge 2010:17). However, Macchia (2003:1120) thinks that the word ‘oral’ does not encompass the written testimonies used by the early church at Azusa Street Revival, so he prefers the term ‘non-academic theology’. Therefore, to understand the Pentecostal religious rhetoric, one needs to analyse the content of prayers, declarations, music and sermons.

Music is a powerful tool used by the AFM in worship. Singing and dancing for the Lord are essential in the AFM in Zimbabwe. Albrecht (1999:159) remarked that: ‘The tone and the words of the [*more meditative*] songs help to move the worshipers into a more “intimate communion”’. Further, ‘The music of the Pentecostal song service … seeks to help usher the congregation into the presence of God’ (p. 143). Also, Warrington (2008:219) argued that ‘Pentecostals expect to experience an intimate relationship with God in which he is felt, and they are moved emotionally’. Laying on of hands is essential, and the Holy Spirit gives directions for missions. The AFM in Zimbabwe focuses on the four-square gospel that emphasises that Jesus saves, Jesus heals, Jesus baptises in the Holy Spirit, and Jesus is coming back again. Woodall (2016) argued that the early Pentecostals embraced a fourfold gospel that emphasised Jesus as Saviour, Sanctifier, Healer and Coming King. Therefore, salvation in Pentecostalism is satisfied when the believers participate in these four areas.

Additionally, in the AFM in Zimbabwe, there is a component of ‘spontaneity’ in the Pentecostal spirituality. There is an assumption that the Holy Spirit directs the church and inspires the believers. As such, the believers wait on the Holy Spirit and desire to function under the spiritual gifts. Speaking in tongues is vital to the AFM in Zimbabwe’s theology and praxis. Chinyemba (1999:49) showed that speaking in tongues helps the believers to be healed and receive automatic blessing and joy. All Bible students in the AFM in Zimbabwe must exhibit the ritual of speaking in tongues (Machingura 2011:18). The prosperity gospel is prominent in the AFM in Zimbabwe, and this has attracted many people to come to church. The prophetic voice has led the Pentecostal churches in Zimbabwe to attract many followers (Sande 2017b:49).

## Apostolic Faith Mission in Zimbabwe theology and the disability theology

Theologies, church doctrines, traditions and beliefs have a way of giving meaning to disability. Analysing the famous theologies of the AFM in Zimbabwe helps to understand how they construct disability in the AFM in Zimbabwe. Therefore, the use and interpretation of biblical texts that relate to disability provide links to how disability is constructed in the AFM in Zimbabwe. Findings from this study showed that there is no clear theology about disability within the AFM in Zimbabwe. Individuals approach issues of disability based on their convictions. One pastor argued that:

‘I rely on the Bible and the leading of the Holy Spirit to judge a situation at hand. At times the Holy Spirit tells me that this disability is the work of the devil.’

The Bible remains authoritative in the AFM in Zimbabwe, but the interpreters are ambivalent when it comes to disability issues. Both the Old Testament and the New Testament present the position and space of persons with disabilities differently. For instance, in the law (Lv 21:18–20) the persons with disabilities are not allowed to reach the Israelite congregation of worship. In the New Testament, persons with disabilities were also marginalised and found outside the synagogues, like the blind man sitting at the beautiful gate (Ac 3:2); it is after healing that the blind man went into the temple.

Accordingly, applying the social model of disability, such ambivalence is a barrier for persons with disabilities because pastors can interpret disability in any particular way. In this case, biblical interpretation is at the centre of denigrating persons with disabilities, if it is left open without an institutional position. All the pastors who participated in this study accepted that the subject of disability is difficult to deal with theologically. They highlighted that there is no subject at Living Waters Theological Seminary (LWTS)^2^ pastoral training curriculum that deals with disability studies. I also conducted a documentary analysis of the AFM in Zimbabwe constitution and found out that they do not have any information relating to persons with disabilities. The LWTS library has no Braille Bible, neither are there facilities like ramps for persons with disabilities to use. Such absence of a curriculum, voluntary programmes for persons with disabilities and facilities is worrisome in this 21st century context. This study could not establish the reasons for such a status quo, but recalls the thrust of the social model of disability which explains that disability does not lie with the individual persons with disabilities, but it is the society that disables them. The AFM in Zimbabwe must empathise with persons with disabilities and deliberately develop structures, programmes and even policies that help them.

The theology of exorcism and deliverance in the AFM in Zimbabwe has an impact on disability. From the author’s observations, many AFM preachers in Zimbabwe, who are predominantly pastors, and lay workers create an impression that disability is the work of the devil. There is a great deal of talk about the devil, and how the powers of darkness cannot rule the believers. One of the services during the conference was dedicated to ‘deliverance and breakthroughs’. What was topical in this preaching and teaching was that the devil is the source and author of all bad things in life. In fact, most of the sermons’ rhetoric reiterates that Satan does not want believers to enjoy a good life and one must thank God because you are not disabled (you can walk, talk and see). The belief is that all life challenges that believers face, like poverty, barrenness, misfortunes and disability, are caused by demons. The preacher declared that any health condition or bad situation that is suspicious and may be the devil’s foul play warrants exorcism and deliverance; the preacher has served notice; and breakthrough is guaranteed for any congregants thus suffering. Therefore, the AFM in Zimbabwe’s theology of exorcism and deliverance categorises disability as caused by evil spirits. Sande (2017c:1) argued that victorious living, breaking poverty and stubborn spiritual vices are the marks of Pentecostal theology in Zimbabwe.

Consequently, the theology of exorcism and deliverance loosely links bad things to the devil, constructing meaning from disability in the AFM in Zimbabwe, and does not give room to explore the complexity of the categories of disability. So, the inference that pastors make during exorcism and deliverance sessions labels persons with disabilities. Consequently, this coincides with the social model of disability viewpoint that society disables persons with disabilities by making them objects of deliverance. Such hermeneutics are suspicious and cannot be tolerated in the community of those who are purported to have received salvation. Using the social model of disability framework as a prescriptive framework, the AFM in Zimbabwe’s pastors need to reinterpret all negative biblical narratives about disability positively. Amanze (2014:264) argued that the Christian theology of disability should articulate that God is for and is on the side of persons with disabilities because they bear the image of God. On the contrary, the process of exorcism and deliverance has negative connotations. In a study from Kenya, Kabue (2011:14) lamented that some exorcisms are abusive, at times involving beating or lashing. Psychologically, to treat persons with disabilities as needing deliverance and exorcism without establishing the causes of disability is spiritual abuse, and the persons with disabilities are at the receiving end.

The theology of demonstration of the power of God is prevalent in the AFM in Zimbabwe. There is a belief that the ‘calling’ upon a pastor must be authenticated by the flow of the power of God during ministry. All the pastors who participated in this study believed that demonstration of power is when miracles happen, especially when the blind see, the lame walk and the deaf hear. Hence, the objects for the demonstration in this case are persons with disabilities. The belief is that disability is an abnormality which needs correction. Hull (2004:11) argued that the existence of persons with disabilities is a continual reminder of fallen humanity, which is imperfect and is hoping to be redeemed. So, in the AFM in Zimbabwe, the restoration of physical ability proves the existence of the power of God. A pastor passionately said:

‘We are a Pentecostal church, we believe in miracles, in this church the blind, lame and deaf used to heal. Where is that God, I tell you today such anointing is still available.’

He further elaborated about the miracles that purportedly followed the Sekuru Chihari ministry. Such sentiments show that the AFM in Zimbabwe has the mandate to restore persons with disabilities and strives to satisfy today the reality of the New Testament scriptures which demonstrate such restoration. The social model of disability helps in this article to see how the theology of ‘demonstration of power’ in a way reduces the human dignity of persons with disabilities. Persons with disabilities are devalued to channels allowing the flow of God and his attestations. In as much as miracles are a reality in the AFM in Zimbabwe, targeting persons with disabilities becomes a snare. Belser (2015:177) suggested that the goal of disability theology is to honour the dignity of persons with disabilities’ lives and to act in solidarity with activists striving for disability justice.

The pastoral ministry must accept that the salvation of the souls of persons with disabilities is essential, more so than their conditions. So, this helps pastoral ministry not to approach disability from a position of pity or sympathy, but perhaps from pastoral care. As such, the AFM in Zimbabwe’s endless prayers towards restoring persons with disabilities and demonstrations of power denigrate the dignity of the persons with disabilities by excluding them from free participation in the religious worship. Demonstrations of power must not disable persons with disabilities, as shown by the social model of disability. But an authentic theology of demonstration of power should foster a spirit of love and belonging, and create a caring community for persons with disabilities. A ‘caring community’ defines the nature of the church and pastoral ministry and is the condition for the proposed transformation. Tools of pastoral care should empower persons with disabilities to experience the Triune God and develop holiness and wholeness. By linking scriptural content with the everyday world and life, pastoral ministry can address the predicament of persons with disabilities and meet their needs. The pastors in the AFM in Zimbabwe, and perhaps the entire Pentecostalism in Zimbabwe, must be versatile in their approach to dealing with the issues surrounding disability.

Healing theology is at the centre of the AFM in Zimbabwe praxis. Gaiser (2010:56) argued that in the Bible, healing is both a communal and a social affair. Over half of the participants from focus groups concurred that the church’s first reaction upon encountering persons with disabilities is anticipation for miraculous healings or eradication of their disabilities through divine intervention. Such an attitude constructs disability as an illness needing treatment. The healing stories of Jesus have served as proof of the moral imperfection of people with disabilities (Grant 1998:77). During the AFM in Zimbabwe conference (August 2016), I heard the preacher calling categorically for persons with disabilities to come for healing prayer. Such a ‘call’ leaves them with no options but to go to the front with others having different type of diseases. So, unconsciously, this practice makes the non-disabled feel that the persons with disabilities need healing. Such biases enhance the marginalisation of persons with disabilities by violating their liturgical freedom. Prayer lines and calls to persons with disabilities are internalised praxis barriers that render persons with disabilities second-class.

A quarter of the persons with disabilities in this study said they feel embarrassed every time they go to the ‘healing line’, and they are not healed. Therefore, formal spiritual declarations by the pastors that ‘today is your day’ of healing create a poor self-image in persons with disabilities. Clifton (2014:213) argued that rather than helping persons with disabilities, ‘the way Pentecostals preach and pray for healing, impacts negatively people who are not healed especially those with a disability’. The framework of the social model of disability provides clues about unjust structures, attitudes and perceptions that society holds against persons with disabilities. Religion plays a significant role in shaping the identity of persons with disabilities (Nzayabino 2005:27), and this practice creates a negative picture of persons with disabilities. The author also observed in the AFM in Zimbabwe that many of these healing prayers targeting the persons with disabilities did not bring about the healing that is claimed by the preacher before the prayer. In this vein, the social model of disability advocates that it is the society (pastorate) that has to change and not the conditions of persons with disabilities. Woodall (2016) argued that in Pentecostalism, while many may receive healing and miracles, other faithful believers remain sick despite much prayer. Unfortunately, these individuals feel isolated and try to hide their disabilities because of embarrassment or the personal feeling of condemnation in a church environment which emphasises miracles and divine healing.

## Apostolic Faith Mission in Zimbabwe liturgical praxis and disability

This section explores the AFM in Zimbabwe liturgical praxis like praying, altar calls and testimonies, songs and music, religious marketing, and how disability is constructed. Most of the marginalisation of persons with disabilities in the AFM in Zimbabwe is secretly reinforced by the liturgical expression toward them in the church. From the author’s participatory observations, the church conferences are usually the most suitable playing fields or grounds in which the ‘us’ and ‘them’ dichotomy is played out in the AFM in Zimbabwe. Typical rhetoric reiterated by the clergy at conferences, especially when praying for the sick and persons with disabilities, is that they should stir up their faith and expect a miracle because today is their day. A third of the focus group participants explained that when such miracles do not happen as preached and promised, they partly blame themselves. Accordingly, this offers a very different and challenging view to the pastoral ministry, not just about the nature of the problem of praying for persons with disabilities, but also about how to integrate human agency and divine agency when miracles fail to happen. Equally important, for today at least, it raises the question as to whether miracles have a role to play in dealing with disability. Eiesland (1994) argued that the Pentecostal churches make one feel they are responsible for the required ‘cure’ as the church’s aim is the ‘normalisation’ of persons with disabilities. In this light, and to correct it, it is vital for pastors to utilise extra-biblical materials as they engage with persons with disabilities – for sensitivity when preaching or doing pastoral duties in the context of persons with disabilities. Pembroke (2009:21) argued that preachers must connect the theology of the text with experiences from scholarly debates coming from disciplines like anthropology, psychology, sociology or philosophy.

Instead, praying for persons with disabilities is either positive or negative, and that increases specific demands and responsibility on persons with disabilities and the congregation. The demand is that persons with disabilities accept prayers and ill-treatment/marginalisation. Also, prayers increase the demand for congregation to accept persons with disabilities rather than a collective working together of PWD, believers and pastors. White (2014) argued that when it comes to the work of Christ in the world and within our lives, there is no difference between the persons with disabilities and the non-disabled. Therefore, pastoral ministry to the persons with disabilities must change and shift from the dichotomy of *us* (pastors) and *them* (persons with disabilities) to the position of ministering *with* the persons with disabilities. Thus, besides the focusing of laying hands on persons with disabilities, the pastoral ministry needs to spend considerable time formulating strategies on how to minister meaningfully to persons with disabilities. Such an approach would provide opportunities for creating safe spaces for persons with disabilities in the church. Assuming responsibility for the persons with disabilities means that ‘the church should be open to the persons with disabilities, to fulfil its call to take care of the disadvantaged and vulnerable’ (Basselin 2011:48). If Basselin’s argument is anything to go by, then the responsibility of the pastors does not only involve the ‘altar call’ and ‘laying of hands’ on the lame, blind and deaf among others, but includes accepting and creating space, creating an enabling environment for persons with disabilities to feel secure and acknowledging them as ordinary human beings.

Testimonies are a crucial component in the AFM in Zimbabwe. They act as the tool for both missions and authenticating the presence of God in the church. Burger et al. (1997:167) argued that most people join the AFM in Zimbabwe because of hearing testimonies. For example, the AFM in Zimbabwe named their national shrine as ‘*Rufaro*’, meaning happiness. This was given as a result of how people witnessed the hand of God and testified publicly to the phenomena. In this study, many testimonies of deliverance and healing were given at the General Conferences in August 2016. The candidates were awarded the time to describe their conditions and how the power of God had moved upon them and also healed them. The congregants would like to respond distinctly because it gives them a sense of serving a mighty God, and many people would cheer and glorify God. In this study, it was hard to explain how emotional testimonies impact persons with disabilities, but I would assume that they would like to receive such miracles and one day testify like the non-disabled. By understanding the implications of the social model of disability, testimonies of the non-disabled put a demand on persons with disabilities; persons with disabilities are pressured to participate in the faith and also to offer testimonies about themselves.

One participant testified that the devil wanted to ‘cripple her’ and ‘make her a useless person’, but glory is to God who makes our triumph. Of course, one could testify and praise God, but to equate disability and suffering, and that God hates it, is overstretching. Therefore, testimonies of what God is doing in the lives of believers create a view that persons with disabilities are suffering because of their condition. Thus, applying the social model of disability in this study, testimonies can be a barrier to persons with disabilities because they fail to account for the needs of persons with disabilities. Further, the consequences of this failure not only apply to pastors – the congregation has a role to play in transforming pastoral ministry to persons with disabilities. By applying the social model of disability perspective, the way non-disabled people speak and explain experiences can form barriers to persons with disabilities. So, the testimonies in the AFM in Zimbabwe encourage persons with disabilities to participate in the church activities but at the same time stereotype persons with disabilities as weak. Further, it may be entirely appropriate for pastors to have the responsibility to teach about and expose invisible barriers – such as unjust structures, attitudinal challenges, ideological issues, demeaning and superiority complexes – that militate against persons with disabilities. Accordingly, there is a link between pastoral ministry and worship in the life of the persons with disabilities. In this case, pastoral care will be the help provided to persons with disabilities in times of emergency and the routine of day-to-day life by lay or ordained Christians.

The singing of certain hymns, using music and dancing are pillars of the AFM in Zimbabwe worship. One outstanding feature I observed during the conference is how the AFM in Zimbabwe use songs as an instrument to communicate their theology about their love for God. Although the songs seemed to make the believers connect with the supernatural world, the content of the message was worth taking note of. The content of some hymns uses physical blindness as a metaphor for spiritual fault, an example being the following AFM in Zimbabwe hymn:

*Mweyamustve WaMwari* [Spirit of God]
*Rega kundipfuura* [Do not pass me]
*Ngandione ndiri bofu* [Let me see I am blind]
*Taurai Izwi rinesimba3* [Speak a powerful word] …^3^

Songs in the AFM in Zimbabwe create a mechanism of stigmatisation by describing ‘physical blindness as spiritual fault’ versus physical sight as spiritual maturity, darkness as abnormality versus light as normality. Such liturgical expressions suggest that there are some actions the AFM in Zimbabwe performs without thinking deeply about the impact it may cause to the person with disability. This is in line with the finding of Earey (2012:11) who contended that the language of some songs and hymns marginalises the persons with disabilities. For example, for persons with disabilities who live in darkness as a normal state, equating darkness with sin has a negative impact. Another liturgical expression I observed during my participant observation is that their phrases, such as ‘lets us raise our hands to the Lord’ and ‘let us close our eyes’, presuppose that everyone has hands, eyes, legs. The author observed that there were no facilities like ramps and sign language interpreters to help persons with disabilities.

Currently, at the time of doing this study in 2017, most churches in Zimbabwe have embarked on religious branding and marketing. The demands for modernity and the information technology age have forced the AFM in Zimbabwe to advertise their spiritual product. As such, in a way, most AFM in Zimbabwe Pentecostal conferences’ advertising has a bearing on disability. The central themes are advertised during the services. The church expects that the lame should walk, the deaf and dumb should speak, and the blind should receive their sight. The author observed that the pastors rhetorically feature the need for miracles in the bulk of their sermons. It would seem to imply that the frustration in the church, because it cannot ‘restore’ the persons with disabilities to what is held to be normal, is the source of the church’s adverse treatment of persons with disabilities in the church. Although marketing the services and sermons for the people to attend, the conferences and service in the AFM in Zimbabwe are good, but the methods used fortify the negative attitudes and beliefs about disability.

One participant who was a male, aged above 40 years, said that:

‘At church, persons with disabilities encounter problems they encounter outside; in fact, such a person will be having layers of disadvantage: from the family, the society and the church with discrimination constituting the most significant percentage of those drawbacks.’

Accordingly, this finding agrees with that of Wolfensberger (1988:15–16) who argued that the marginalisation of persons with disabilities comes when people view them as objects of charity needing healing. He further pointed out that the Christian community devalues persons with disabilities by viewing the disabled as the ‘other’ or ‘alien’. Therefore, that the Christian community sees disability as a temporary affliction that must be endured to gain heavenly rewards. Mutswanga, Makoni and Chivasa (2015:174) argued that ‘The mainstream thinking in the Pentecostal circles in Zimbabwe have turned a blind eye to the issues of stigma to the persons with disabilities’. It is problematic in this study to ascertain how much the societal perspectives outside the church affects the persons with disabilities. In describing the social model of disability, Barnes and Mercer (2010:163) explain that society is responsible for disabling persons with disabilities. Perhaps the problem of adverse treatment is not limited to the AFM in Zimbabwe. In the context of the AFM in Zimbabwe, the social model of disability comprehends the effect of pastoral ministry and care of persons with disabilities. The implication is that the AFM in Zimbabwe should take a leading role in efforts to correct these negative attitudes towards persons with disabilities, because most of the attitudes and sentiments have their roots in religious doctrines.

## Conclusion

The intended goal of this article was to explore the AFM in Zimbabwe pastoral ministry’s response to disability. The article explored the practices and processes through which disability in the pastoral ministry of AFM in Zimbabwe is constructed. The findings from this article showed that there are invisible barriers that militate against persons with disabilities, and both the pastors and the congregation need to seriously consider the human rights of persons with disabilities and their divine worship space. Inevitably, the pastoral ‘divine solutions’ and ‘triumphalist messages and teachings’ are ‘prescriptive’ and ineffective in reducing ‘the disability prevalence in Zimbabwe’. Thus, the pastoral ministry should be ‘one efficient vehicle’ with which the church can care for and ‘transform persons with disabilities’. Pastors should break the glass ceiling by expecting pastors to minister better and more effectively, creating a safe space for persons with disabilities. A caring community should be the nature of both the AFM and the pastoral ministry responsible for meeting the needs of the persons with disabilities.

## Footnotes

1 PhD in Religion and Social Transformation, University of KwaZulu-Natal, fieldwork data collected in Harare, Zimbabwe, in 2016.

2 Living Waters Theological Seminary is the AFM training wing. It was established in 1974 and has attracted a considerable number of students from Southern Africa.

3 Nziyo Dze Chipositori AFM Shona Hymn: *Ishe Ndinzwe Nokufara*.
